# Biomechanical Finite Element Method Model of the Proximal Carpal Row and Experimental Validation

**DOI:** 10.3389/fphys.2021.749372

**Published:** 2022-01-24

**Authors:** Rafael Marqués, Juan Melchor, Indalecio Sánchez-Montesinos, Olga Roda, Guillermo Rus, Pedro Hernández-Cortés

**Affiliations:** ^1^Department of Structural Mechanics, University of Granada, Granada, Spain; ^2^Department of Statistics and Operations Research, University of Granada, Granada, Spain; ^3^Instituto de Investigación Biosanitaria, ibs.GRANADA, Granada, Spain; ^4^Excellence Research Unit “Modeling Nature”, University of Granada, Granada, Spain; ^5^Department of Anatomy and Human Embryology, University of Granada, Granada, Spain; ^6^Department of Structural Mechanics, University of Granada, Granada, Spain; ^7^Department of Surgery, University of Granada, Granada, Spain

**Keywords:** FEM, biomechanics, scapholunate ligament, experimental, computational

## Abstract

The Finite Element Method (FEM) models are valuable tools to create an idea of the behavior of any structure. The complexity of the joints, materials, attachment areas, and boundary conditions is an open issue in biomechanics that needs to be addressed. Scapholunate instability is the leading cause of wrist pain and disability among patients of all ages. It is needed a better understanding of pathomechanics to develop new effective treatments. Previous models have emulated joints like the ankle or the knee but there are few about the wrist joint. The elaboration of realistic computational models of the carpus can give critical information to biomedical research and surgery to develop new surgical reconstructions. Hence, a 3D model of the proximal carpal row has been created through DICOM images, making a reduced wrist model. The materials, contacts, and ligaments definition were made *via* open-source software to extract results and carry on a reference comparison. Thus, considering the limitations that a reduced model could carry on (unbalanced forces and torques), the stresses that result in the scapholunate interosseous ligament (SLIL) lead us to a bones relative displacement, which support the kinematics hypothesis in the literature as the distal carpal row moves as a rigid solid with the capitate bone. Also, experimental testing is performed, successfully validating the linear strength values of the scapholunate ligament from the literature.

## 1. Introduction

Scapholunate dissociation is the most frequent pattern of carpal instability (57% of total wrist injuries) and is related to a lesion that affects the scapholunate interosseous ligament (SLIL) (Pappou et al., 2013; Elsaftawy et al., 2014). Hence, the scapholunate ligament is considered the primary stabilizer of the scapholunate joint (Garcia-Elias et al., 2006). When there is an injury of the scapholunate ligament, the junction between the scaphoid and lunate becomes unstable; the more unstable it becomes, the larger the damaged ligament portion. The secondary stabilizers (as radio-scaphoid-capitate or scaphoid-trapezium-trapezoid ligaments) become progressively incompetent due to fatigue (Kitay and Wolfe, 2012). This loss of joint relationship favors the development of radiocarpal and midcarpal osteoarthritis (Harrington et al., 1987; Watson et al., 1997).

The dramatic evolution of the lesion that we expose and the absence of efficacious treatment have promoted the experimental studies of kinetics and kinematics of the carpus in order to improve the knowledge of carpal instability and look for alternative treatments. Finite Element Method (FEM) models are helpful tools used in engineering that allow us to study complex areas as a first step, giving us an idea of the necessity of clinical investigation.

This study aims to recreate a functional wrist model *via* the FEM to evaluate the stabilizing role of the SLIL and the effect of its failure, and the possibility of recreating different pathologic situations to better pathomechanics understanding by clinicians. First, a study of the geometry is implemented, using actual cadaveric wrist specimen CT scans, reconstructing the volumes for each bone, and the cartilages models. After that, the materials are implemented, evaluating the structural importance of the cancellous bone. Finally, and, attending to the study of Johnson et al. (2013), Stoesser (2016), Werner et al. (2011), and Bryce (1896), a reduced carpal model is constructed, including only the radius, ulna, scaphoid, lunate and the capitate bone, since these are the ones responsible for the stability of the scapholunate zone. Unidimensional models of the ligaments are implemented in the complex and a 3D model for the SLIL. The study would be based on the data on kinematics and previous experiences of several authors centered on the studied area, analyzing the stresses and strains in each bone and with the final objective of removing the SLIL from the model to evaluate the behavior of the complex in its absence.

Finally, experimental validation of the ligament stiffness is made using a tensile testing machine and cadaveric SLIL samples. The idea is to combine accurate data from cadaveric specimens with the possibilities of a numerical simulation, studying the mechanic response of the structure, in order to gather more information for future studies of the SLIL instabilities and carpal structure in general.

## 2. Materials and Methods

### 2.1. Theoretical Background

The wrist joint complex consists of 8 carpal bones, divided into two rows and arranged between the distal radius/ulna and the proximal metacarpals. In this study, we are studying the behavior of the SLIL, which is the ligament between the lunate and scaphoid bones. That is why our investigation will focus on the radius (ulna does not affect the SLIL joint, it does with the triquetrum/pisiform), the proximal row (especially in these two bones), and one of the distal row's bones, the capitate. Articular cartilage (Mohd Nazri Bajuri, 2013), composed of hyaline cartilage, is mainly located at the ends of long bones, and it is the one involved in our research. The ligaments function as constraints (mainly as tensile resistance) and stabilizers on the wrist motion (Mohd Nazri Bajuri, 2013). We can divide the ligaments complex into two main groups: intrinsic and extrinsic ligaments. Intrinsic ligaments are those located between adjacent carpal bones. On the other hand, the extrinsic ones fix the carpal bones to the radius and ulna. As it is the main character of this investigation, a more complex view of SLIL is made, following the definitions given by Stoesser (2016).

In order to evaluate stresses and strains in the SLIL, a continuum mechanics interpretation of the material can be made. Several authors can be followed in order to decide the best material type for each part involved in the study (2002; Gíslason et al., 2010; Bajuri et al., 2012; Gíslason and Nash, 2012; Mohd Nazri Bajuri, 2013). Bones (both cortical and cancellous bone) can be defined as “Elastic Linear Isotropic Materials” described by Young's Modulus (E) and Poisson's ratio (**ν**). Cartilages can be expressed in terms of hyperelastic (or green elastic material), a type of constitutive model for ideally elastic material for which the stress-strain relationship derives from a strain energy density function. The cited authors make use of the Mooney-Rivlin formulation based on the material parameters (C_1_), (C_2_), and Bulk modulus (***K***). For intrinsic and extrinsic ligaments, an hyperelastic material can be chosen, although many authors use linear spring link elements attached to their bone insertion area and described by their linear elastic constant ***k***.

The finite element (FE) method is a computational numerical analysis technique, which is combined with computational modeling to obtain stress and strain patterns in modeled bodies, using the continuum mechanics theory and has been widely used as solutions to engineering problems. A variety of shapes and models can be analyzed using the FE method. Hence, using the material definition made below, a FE model is the tool chosen for the analysis of the problem. The software chosen for this computational model is Febio2.0 (Maas et al., 2012; 2017), an open-source non-linear FE solver that is specifically designed for biomechanical applications. It offers modeling scenarios, constitutive models, and boundary conditions relevant to many research areas in biomechanics. The necessary information for the model is analyzed below in the logical sequence of any FEM problem.

### 2.2. Computational Model

#### 2.2.1. Defining the Geometry

The geometrical representation of the model was obtained from CT scans of the average human wrist. Nowadays, we can find several programs that can carry out image processing and segment the scans to create a three-dimensional surface. In our case, an open source software, only for research use, from the “Center for Information Technology Renato Archer” named Invesalius (v3.1.1) (De Moraes et al., 2011) was used.

Many authors have determined correlations between the mechanical properties of the SLIL and many other variables (Bone Mineral Density, forces between carpal bones, loads applied to the ligament) (Berger et al., 1999; Johnston et al., 2004) pointing no significant differences between gender or age. This literature conclusion supports our decision of using only one subject for the 3D model elaboration. As gender and age can cause a variation in the bones properties that these authors consider, and there exists a variability in bone sizes between population, a further model considering re-scalation of bones to represent different gender (de Roo et al., 2019) and ages (Kaawach et al., 2001) would be necessary.

The scans were taken from the wrist of the subject (women, 48 years old), ranging from the distal end of the two forearm bones, radius and ulna, to the proximal third of the metacarpals. The total length of the scans was 58 mm. The resolution of the scans was 283 μm in-plane, and the space between slices was 2 mm. Using the manual tools of the software, a masking process is made bone by bone, obtaining simple rough models. This masking process is also useful in differentiating between cortical and cancellous bone because the cancellous part has a lower image density and, consequently, contrast. The importance of high-quality segmentation in multi bone modeling is an important task, as the congruence of the articulating surfaces will play an important role in the contact formulation. Rough edges on surfaces can cause penetration of node points, causing numerical instability and convergence problems. Hence, post-process sculpting work was carried out, so the geometry gets more similar to the real bone (Ebrahimi, 2012).

**Figure 1:**
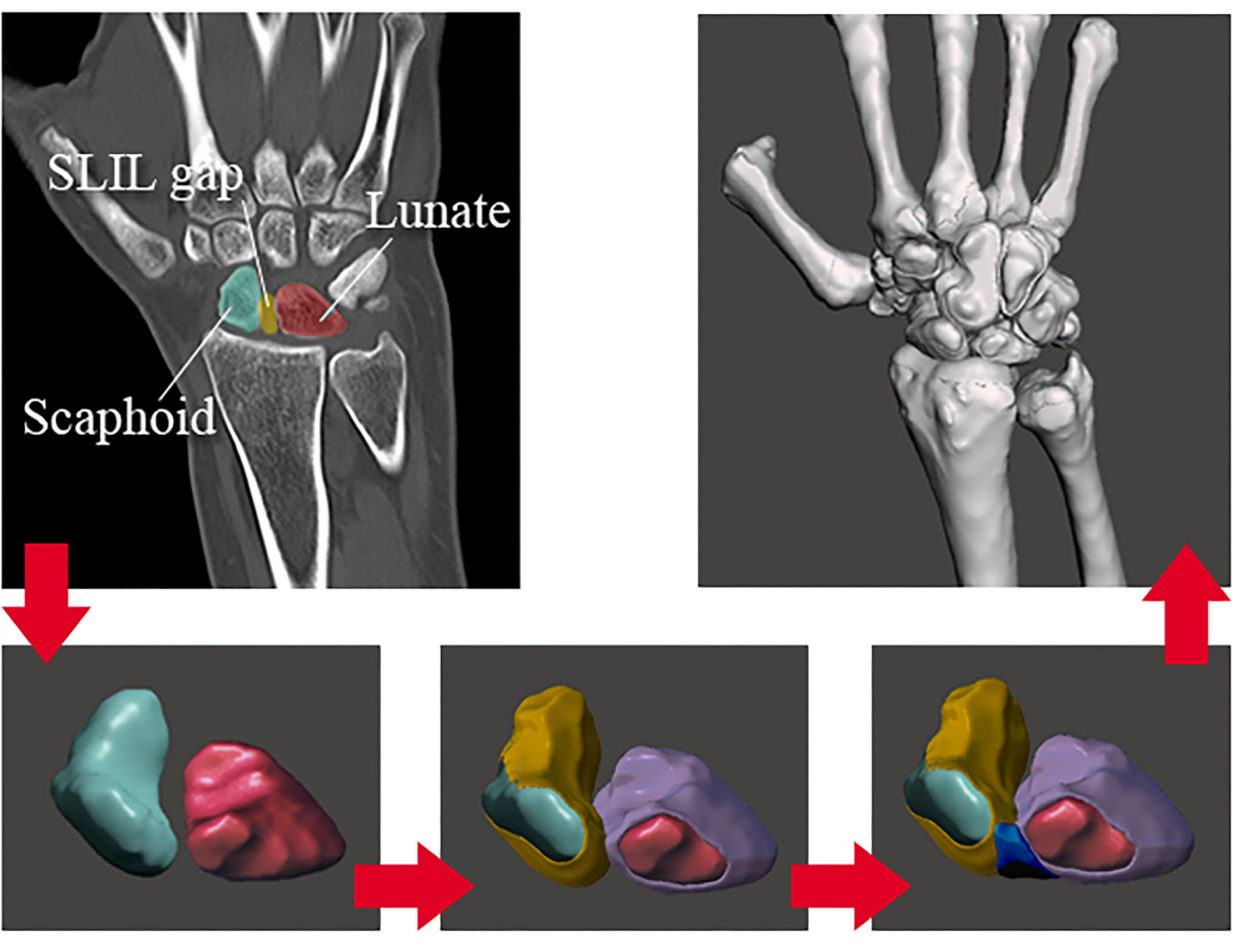
DICOM images reconstruction process.

The process for extracting the cartilages (and SLIL) 3D models follows a similar study planning but uses the bone models (already created) and boolean geometrical operations (Mohd Nazri Bajuri, 2013). The process followed is shown in Figure 1.

This first 3D model from the carpal structure opens up the path for future biomechanical designs. The results obtained in this study can make the first iteration for a validation process. Thus, future study comparing kinematics between our model and actual data from literature/experimental values would be necessary.

#### 2.2.2. Meshing Process

By using the software Autodesk®(San Rafael, California, CF, United States) Meshmixer^TM^ (Autodesk®, 2017), on its educational version, a simple triangular mesh has been elaborated, attending to some basic rules in order to avoid convergency errors in our results. The rules to be considered in the meshing process are: avoid slender elements (one dimension considerably larger than the other); it is recommended to make all the elements of the approximately same size; mesh refinement in the areas which present discontinuities; the refinement must be gradual with adjacent elements of not too dissimilar size; mesh refinement must balance accuracy with problem size. The software has tools that let us objectively validate these requirements (refer to section 3, “Results”).

After the triangular 2D mesh is done and the element triangle shapes accomplish the previous rules, we had to transform this 2D mesh into a 3D one. According to the bibliography and the background, we can find about FEM models in the biomedical area (Carrigan et al., 2003; Marai et al., 2009; Gíslason et al., 2010; Gíslason and Nash, 2012; Mohd Nazri Bajuri, 2013), there are two main types of elements that have been widely used: tetrahedral and hexahedral. Hexahedral elements were less efficient than tetrahedral in contact analysis due to the high number of nodes (more tendency to distort). Despite tetrahedral elements having less DOF than the hexahedral, they are better for curve modelization. Thus, the type of elements chosen for the model is Tetrahedral Elements with four nodes.

FEBio (Maas et al., 2012; 2017) includes 2D to 3D mesh conversion tools. This software is “TetGen,” by the Weierstrass Institute for Applied Analysis and Stochastics (WIAS).

#### 2.2.3. Materials Modeling

##### 2.2.3.1. Bone

The cortical and cancellous bones were modeled using linear, isotropic material properties with Young's modulus of 18 and 100 MPa, respectively. The Poisson's ratio values used were 0.2 for cortical bone and 0.25 for cancellous bone. These values were taken from the literature (Gíslason et al., 2010; Gíslason and Nash, 2012). Nearly all FE studies on joint mechanics have modeled bone as a linear isotropic material, but many references have shown the idea of treating the bone as a solid, ignoring the role of cortical/cancellous bone. In this study, the first option has been chosen, considering the bone as a linear elastic but, in order to evaluate the importance of the cancellous bone in front of the cortical one (due to the lesser stiffness value in the cancellous), a reduced model is made (section 3). The aim is to decide if we can obviate the cancellous bone and make a single piece for each bone with only cortical material.

##### 2.2.3.2. Soft Tissue

The cartilage was modeled using hyper-elastic material properties, as described previously. Li et al. (2007) proposed the parameters making up the curve should be C_10_ = 4.1 MPa and C_01_ = 0.41 MPa for a two-parameter Mooney-Rivlin material. Attending to the definitions made by Bower (2009), the Mooney-Rivlin model is a special case of the generalized Rivlin model (also called polynomial hyperelastic model), which has the form:


(1) $$\begin{array}{l} W=\Sigma_{p,q=0}^{N}C_{pq}{\left(\bar{I_{1}}-3\right)}^{p}{\left(\bar{I_{2}}-3\right)}^{q}+\Sigma_{m=1}^{M}D_{m}{\left(J-1\right)}^{2m} \end{array}$$

With C_00_ = 0, where C_pq_ is material constants related to the distortional response and D_m_ are material constants related to the volumetric response. For a compressible Mooney-Rivlin material, *N* = 1, C_01_ = C_2_, C_11_ = 0, C_10_ = C_1_, C_01_ = C_2_, M_1_. The resulting equation corresponds to ours, given by the FEBio Theory Manual (2017).

On the other hand, for obtaining the Bulk Modulus of the articular cartilage, we followed the research by Little et al. (1986), who gave experimental obtained values of Young's modulus and Poisson ratio. We know that the relation between these parameters and the bulk modulus is:


(2) $$\begin{array}{l} K=\frac{E}{3\left(1-2\nu \right)} \end{array}$$

Little et al. assume that: *E* = 11.6 MPa and ν = 0.4 for articular cartilage (Little et al., 1986). Introducing these values in Equation (2), we obtain that the bulk modulus of the articular cartilage is *K* = 19.3 MPa. This value is inside the range of values that Vidal-Lesso et al. (2014) consider as possible ones for this modulus on the cartilage.

The SLIL, as we commented, will be modeled as a hyper-elastic Mooney Rivlin two-parameter material, being also necessary for the C_1_ and C_2_ constants and the bulk modulus. The constants will be the ones obtained by Weiss et al. (2002). Hence, the author shows that the constants have average values of:C_1_ = 832.4 · 10^-6^ MPa and C_2_ = 11.05 · 10^-6^ MPa. On the other hand, for the bulk modulus, Weiss gives a “Bulk modulus/Shear modulus” ratio of 1,000 and also shows the next relation: μ= C_1_ · C_2_, obtaining that the bulk modulus of the ligament is: ***k*** = 9.19802 MPa.

The ligament modeling will be as explained previously (linear spring elements) attending to Table 1. In section 3, we are making an experimental validation of the mechanical properties of the SLIL, comparing the axial test results to the cited table.

**Table 1:** Ligaments stiffness for a spring linear element modelization (Mohd Nazri Bajuri, 2013).

**Ligament**	**Connection 1**	**Connection 2**	**Stiffness (N/mm)**
Dorsal intercarpal	Capitate	Lunate	150
Dorsal intercarpal	Capitate	Scaphoid	150
Dorsal scapholunate	Scaphoid	Lunate	230
Long radiolunate	Lunate	Radius	75
Radial arcuate	Capitate	Scaphoid	40
Radial collateral carpal	Radius	Scaphoid	10
Radioscaphocapitate	Radius	Capitate	50
Short radiolunate	Radius	Scaphoid	75
Volar Radioscapholunate	Radius	Scaphoid+Lunate	50.75

#### 2.2.4. Boundary Conditions

For our case, there are two types of BC's: the fixed displacements/rotations for the proximal zone of the radius and the prescribed motions for the carpal row. The fixed conditions affect the bottom surface of the radius. Hence, translational (x, y, z) and rotational (u, v, w) degrees of freedom are zero.

The wrist is a hypermobile joint with multi-planar motions. The most common movements are radial-ulnar deviation (RUD), flexion-extension (FE), and pronation supination (PS), and the possible combinations between them. The PS motion does not involve the scapholunate joint on it; that is why it will not be simulated on the computational model of this study but will be described below. The average range of motion on each case is (Table 2):

**Table 2:** The average range of motion on the main hand movements.

**Wrist motion**	**Normal range of motion**
Flexion-extension	Flexion: 65–80° Extension: 55–75°
Radioulnar deviation	Radial deviation: 15–25° Ulnar deviation: 30–45°
Forearm pronation-supination	Pronation: 60–80° Supination: 60–85°

**Figure 2:**
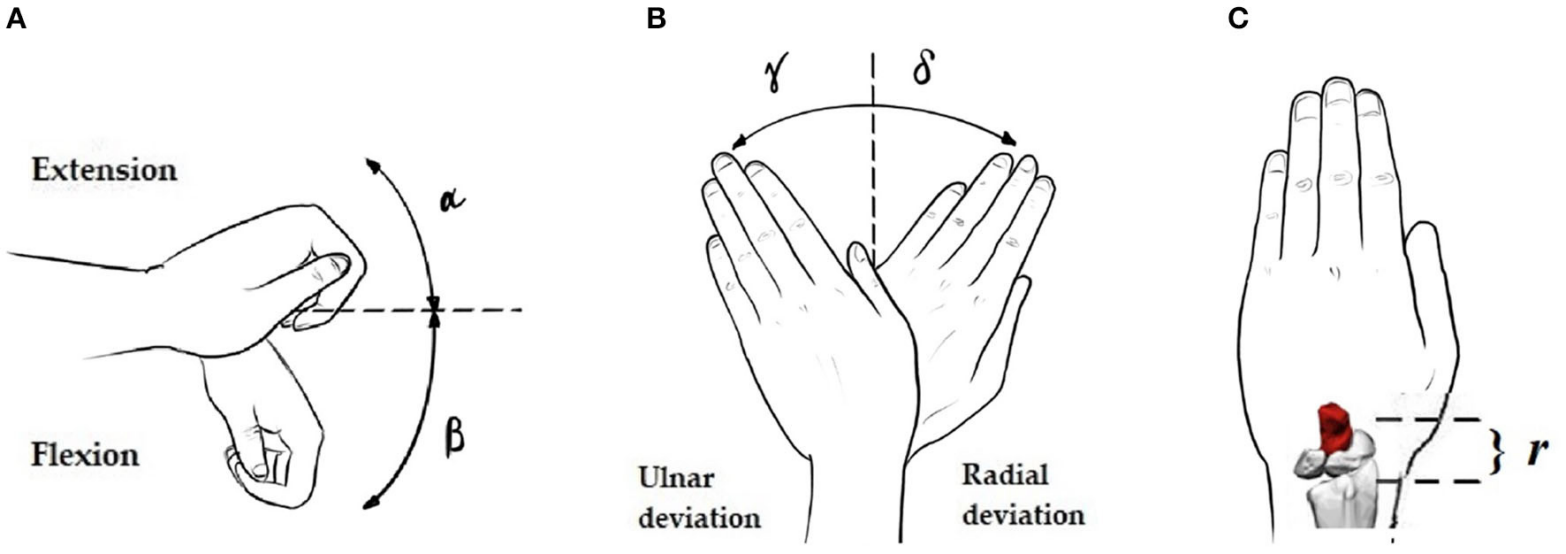
Necessary angles and distances for the rotation-displacement conversion: **(A–C)** Extension angle (α), Flexion angle (β), Ulnar (γ) and Radial (δ) deviation angles, and radius-applied displacements node distance (*r*).

The motion of the wrist will be emulated by prescribed motions applied on the capitate bone to transmit it to the scapholunate complex, translating these rotations into cartesian displacements (Figure 2).

Thus, according to the reference system in our model, flexion/extension angles will traduce in y and z displacements and deviations in x and z displacements. These values are shown in Table 3.

**Table 3:** Displacement values to be applied as prescribed in capitate bone.

**Extension**		**Flexion**		**Radial deviation**		**Ulnar deviation**		
**y**	**z**	**y**	**z**	**x**	**z**	**x**	**z**	
−17.58	−11.20	18.50	−13.56	−6.635	−1.169	11.80	−4.01	[mm]
−0.017	−0.011	0.018	−0.013	−0.007	−0.001	0.012	−0.004	[m]

### 2.3. Experimental Validation

For the elaboration of validation through experimental results, actual human cadaver scapholunate ligaments samples were tested in a tensile testing machine. It was necessary to execute the tensile testing machine's design, construction, and software for tension/compression tests in biomaterials. In addition to this task, design work and 3D printing were carried out for the realization of it.

We tested six cadaver scapholunate ligament samples with a non-degenerative condition (three men of 58, 62, and 65 years old; three women of 60, 62, and 63 years old) to determine the linear stiffness by uniaxial loading. As our particular case of study was the scapholunate ligament, the idea was to add more data about the mechanical behavior of this tissue and, as follows, corroborate the values from Mohd Nazri Bajuri (2013). At the same time, calibration of our self-made axial press with the literature values was implemented, giving us the confidence to use it in future studies.

## 3. Results

### 3.1. 3D Real Geometry Acquisition

**Figure 3:**
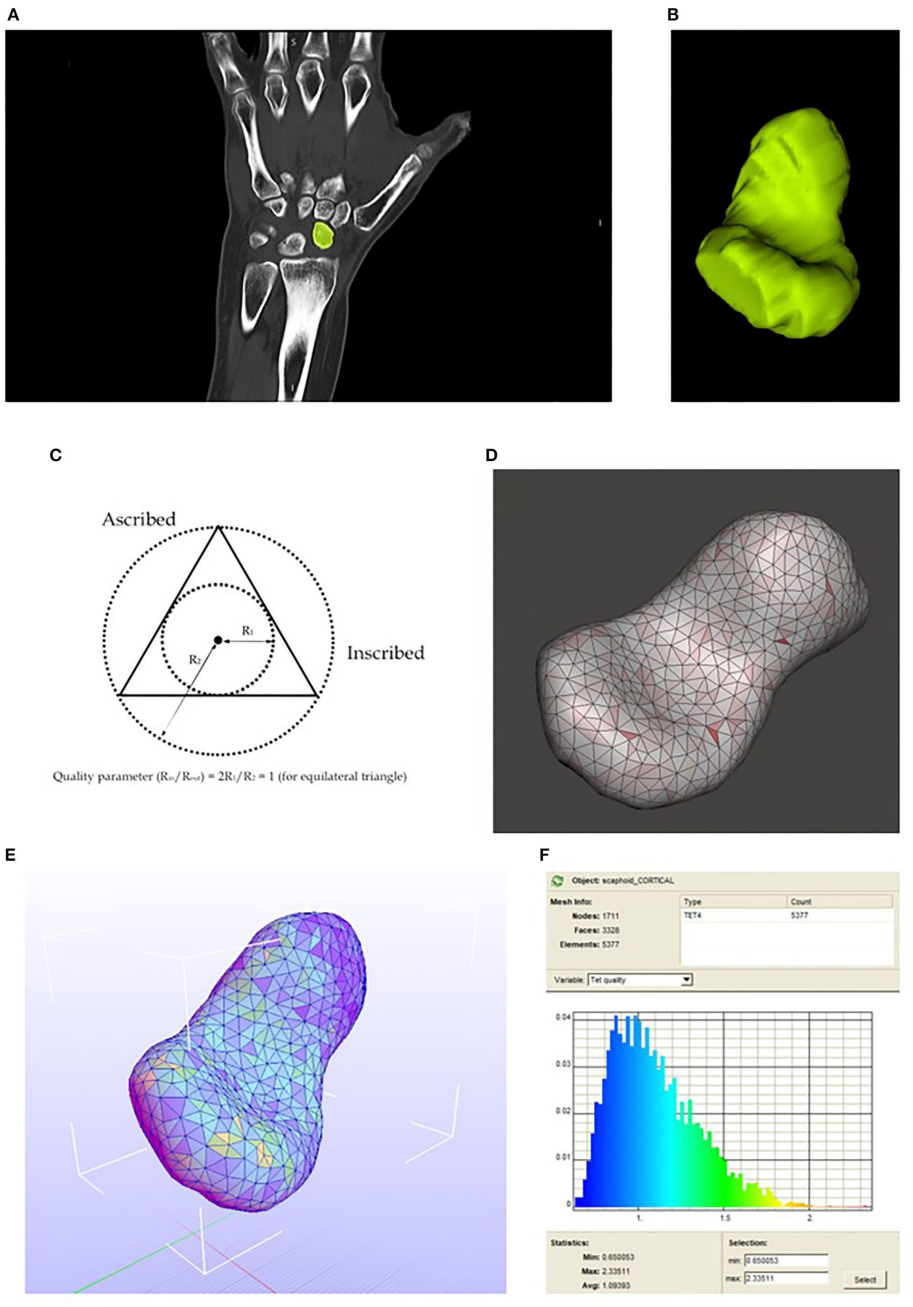
**(A)** Mask drawing in one of the CT scan slices. **(B)** Resulting 3D model. **(C)** Aspect ratio concept. **(D)** 2D meshing. Aspect ratio visualization. **(E)** Scaphoid meshed with tetrahedral elements, the colors in each element match with the Tet quality on the right. **(F)** Tetrahedral elements quality spectrum.

We can see an example of the process in Figures 3A,B showing the masking process in the CT scan view with more resolution, the Coronal plane in our case.

### 3.2. Mesh Validation: Aspect Ratio

With Meshmixer, we can represent the aspect ratio (ratio between the inscribed and ascribed circle) of each element which is an excellent parameter to evaluate if our elements are similar or diverge from equilateral triangles (if aspect ratio approximate to the unity, the element is close to being an equilateral triangle). The result of the meshing process representing the aspect ratio is shown below in Figures 3C,D.

### 3.3. Mesh Transformation: 2D to 3D

The TetGen (Si, 2015) conversion work is directly executed in Febio with a simple option. The result is shown below in Figures 3E,F.

**Figure 4:**
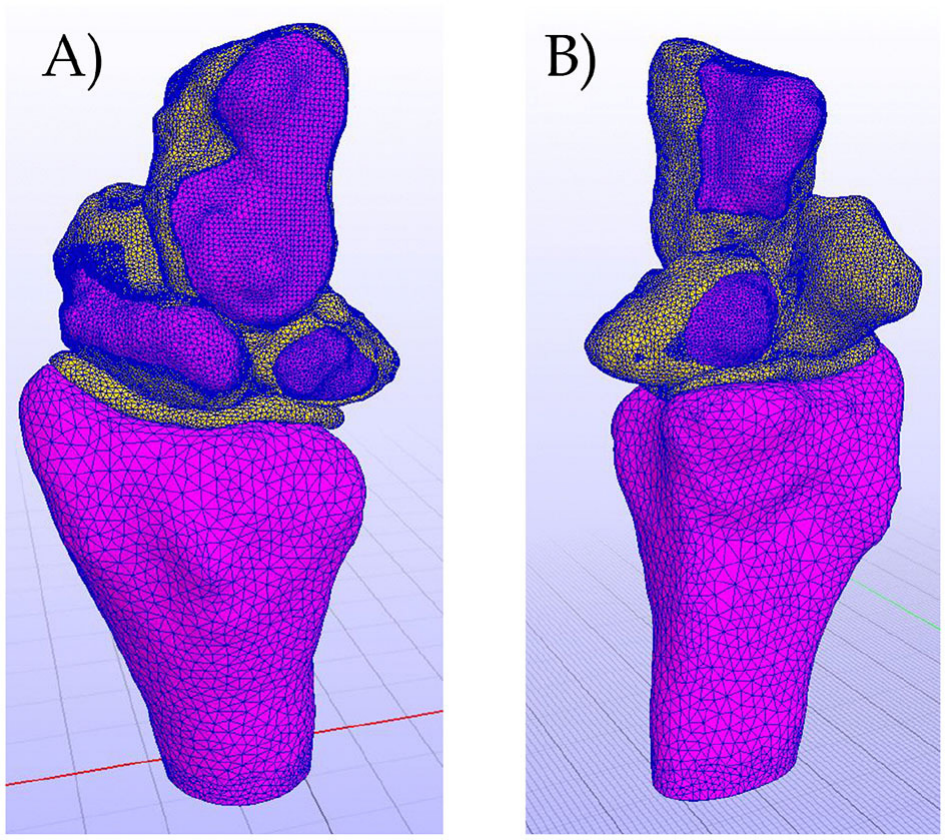
**(A)** Complete meshed model. Dorsal view. **(B)** Complete meshed model. Volar view.

Thus, the final result of the complete bones and cartilages 3D meshed model is shown in Figure 4.

### 3.4. Cancelous Bone Influence in Reduced Model

A reduced model of the distal radius in the face of two loads has been evaluated. The model includes the cartilage of the radius, the cancellous and the cortical parts of it, and, as a boundary condition, it is fixed (both displacements and rotations) in the proximal zone.

The loads are placed in the two cavities of the scaphoid and lunate, and they have unity as value. The values for the materials modelization are the ones shown previously for cancellous and cortical bone.

**Figure 5:**
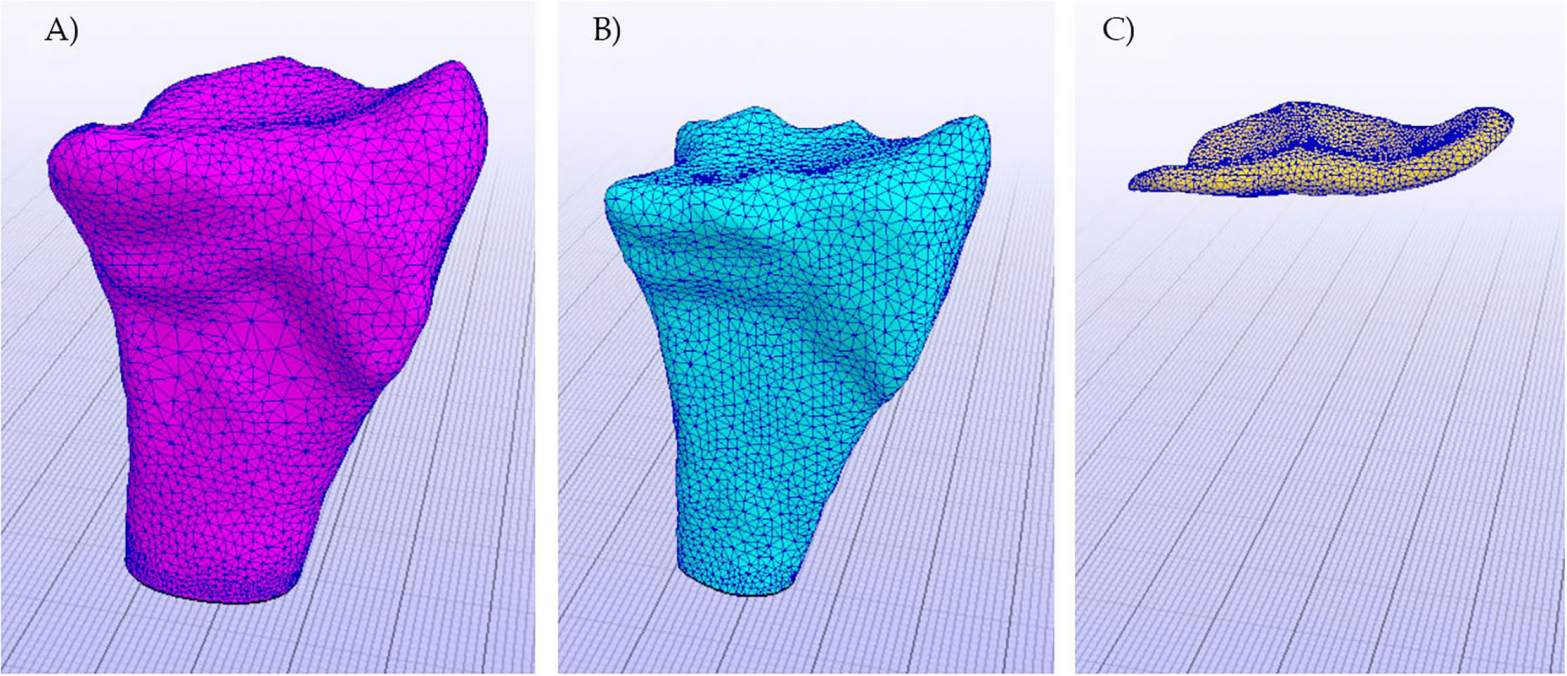
Objects of the model already meshed. **(A)** Cortical bone, **(B)** Cancelous bone, and **(C)** Cartilage.

**Figure 6:**
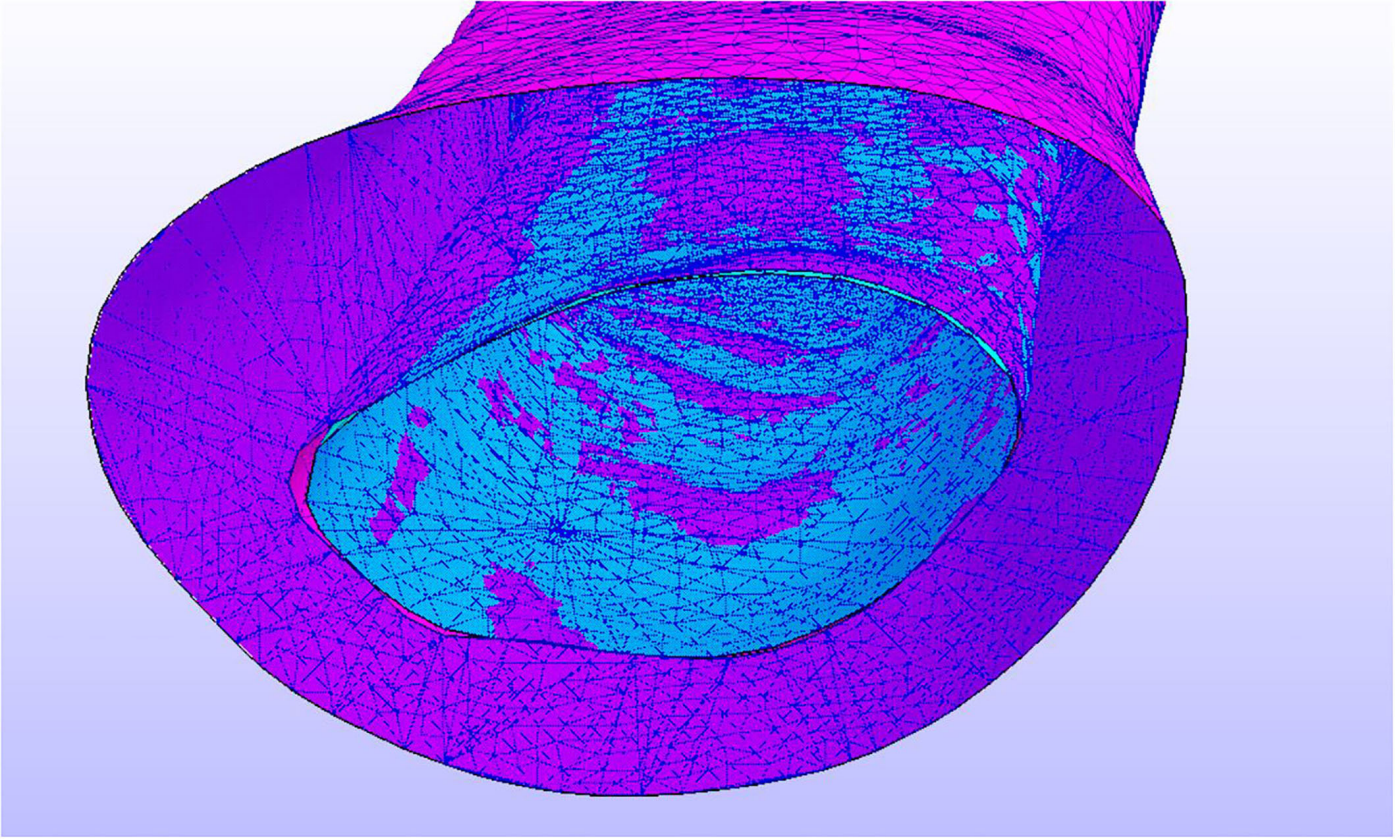
In blue, the area in which the BC's (fixed displacements and rotations) have been applied.

Next, some figures are shown to see several important aspects of the modelization work (Figures 5, 6).

The results were significantly crucial for our work because the hypothesis of ignoring the cancellous part of the bone is validated, and, in our complete model, the bones can be considered one single cortical bone. The results are shown below, and some considerations have been made to argue the previous conclusion.

**Figure 7:**
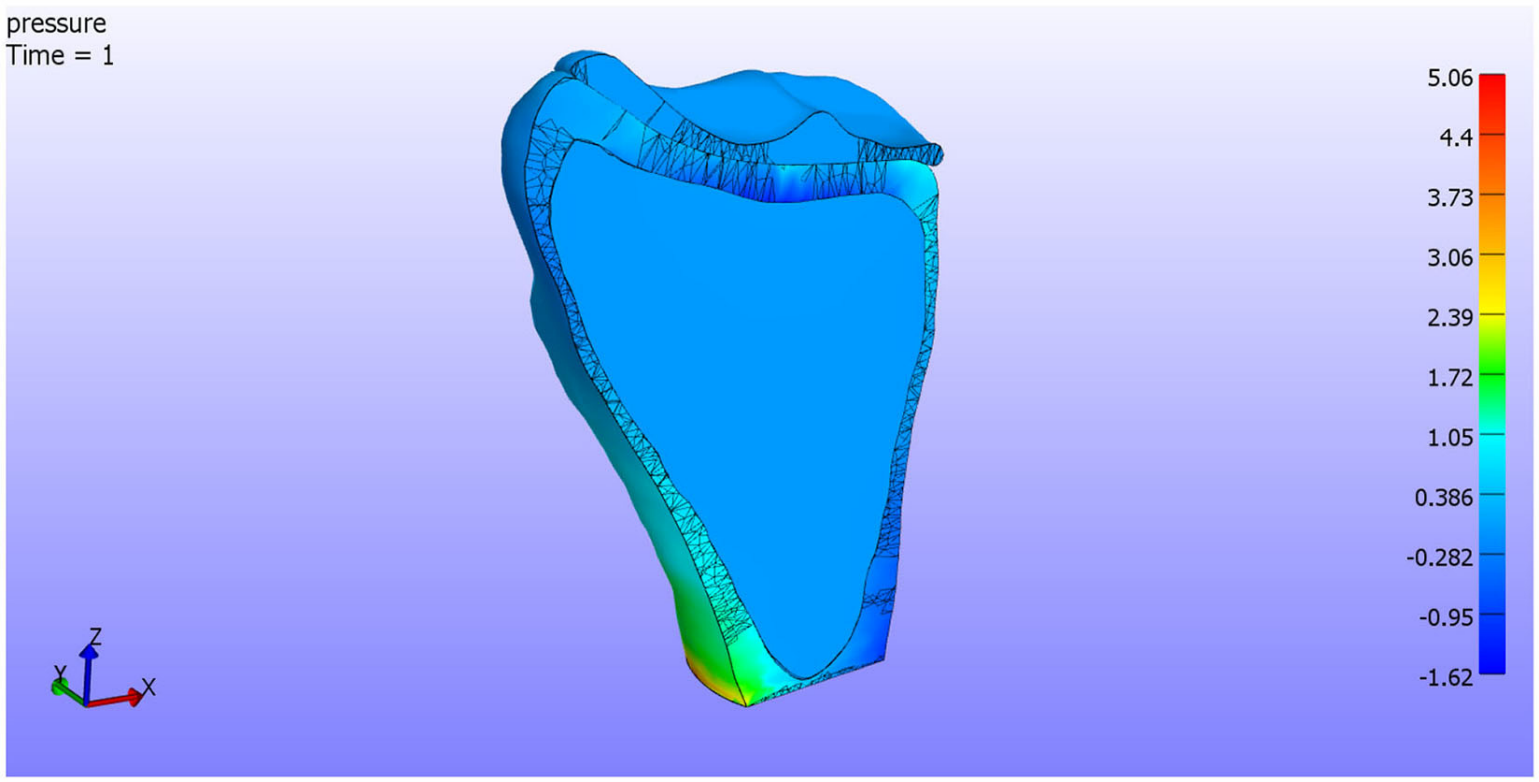
Pressure. Cut by XZ plane.

In Figure 7, we see the pressure on the complex. As is shown, stresses do not affect the cancellous part of the bone, distributing along the cortical part. We can now accept the hypothesis of modeling the bones as a single cortical piece.

**Figure 8:**
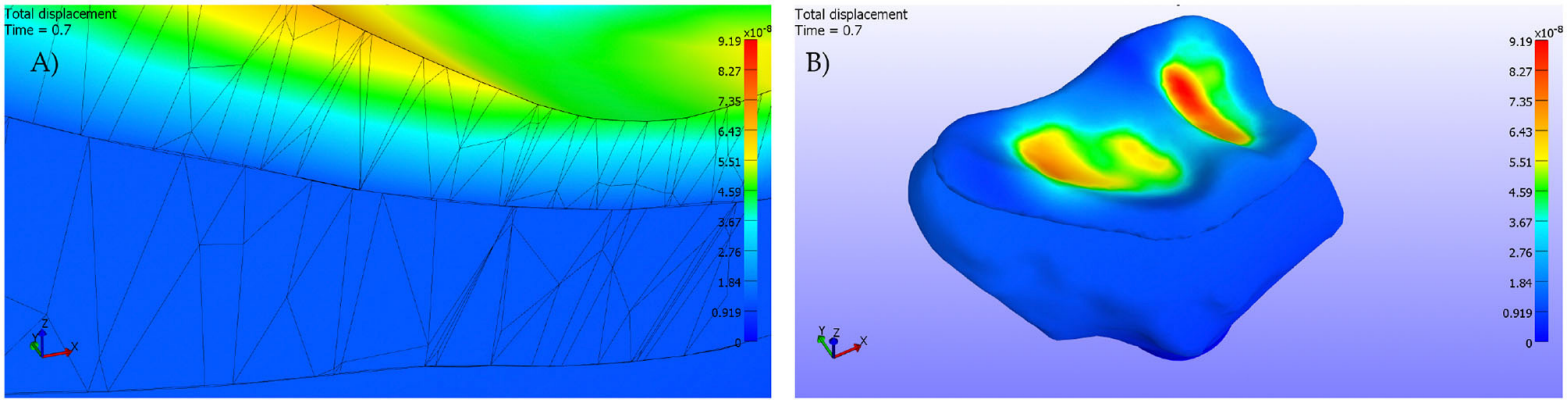
**(A)** Detail. Cut by XZ plane. **(B)** Pressures applied on the cartilage surface.

On the other hand, in Figure 8, we see the total displacements. In Figure 8B, we can see that the displacements are concentrated in the loaded areas, the cartilage support all the deformations. In Figure 8A, a detail of the contact between the cortical bone and the cartilage is shown. The cortical bone is not affected by the displacements as it is more rigid than the cartilage.

### 3.5. Experimental SLIL Young Modulus and Poisson Ratio

The Hospital San Cecilio (Granada) axially tested six human SLIL samples. We can observe close values between the experimental results and the ones in the literature (refer to Tables 4, 5).

**Table 4:** Linear strength modulus obtained for the six human scapholunate interosseous ligament (SLIL) samples.

	**Experimental testing**					
**Sample**	**I**	**II**	**III**	**IV**	**V**	**VI**
k [N/mm]	111.38	24.44	114.12	35.36	90.29	53.44
Mean/Std.	71.50/39.00					

**Table 5:** Literature values for the SLIL's linear strength modulus.

	**Literature**		
**Sample**	**Nikolopoulos et al., 2011**	**Wayne and Tremols, 2019**	**Eschweiler et al., 2016**
k [N/mm]	25–36	66	50–150
Mean/Std.	65.50/34.70		

As we will see, the validation seems to be valid, as it concords with literature values. In this manner, our SLIL Young's modulus has a value of *E* = 4.89 MPa. Thus, introducing these values in Equation (2), we obtain that the Poisson's ratio for the scapholunate ligament is: ν= 0.41 MPa, close to the value in cartilages. We know that the behavior of both is similar, so these values seem to adjust to reality.

### 3.6. Intact SLIL

#### 3.6.1. Extension

**Figure 9:**
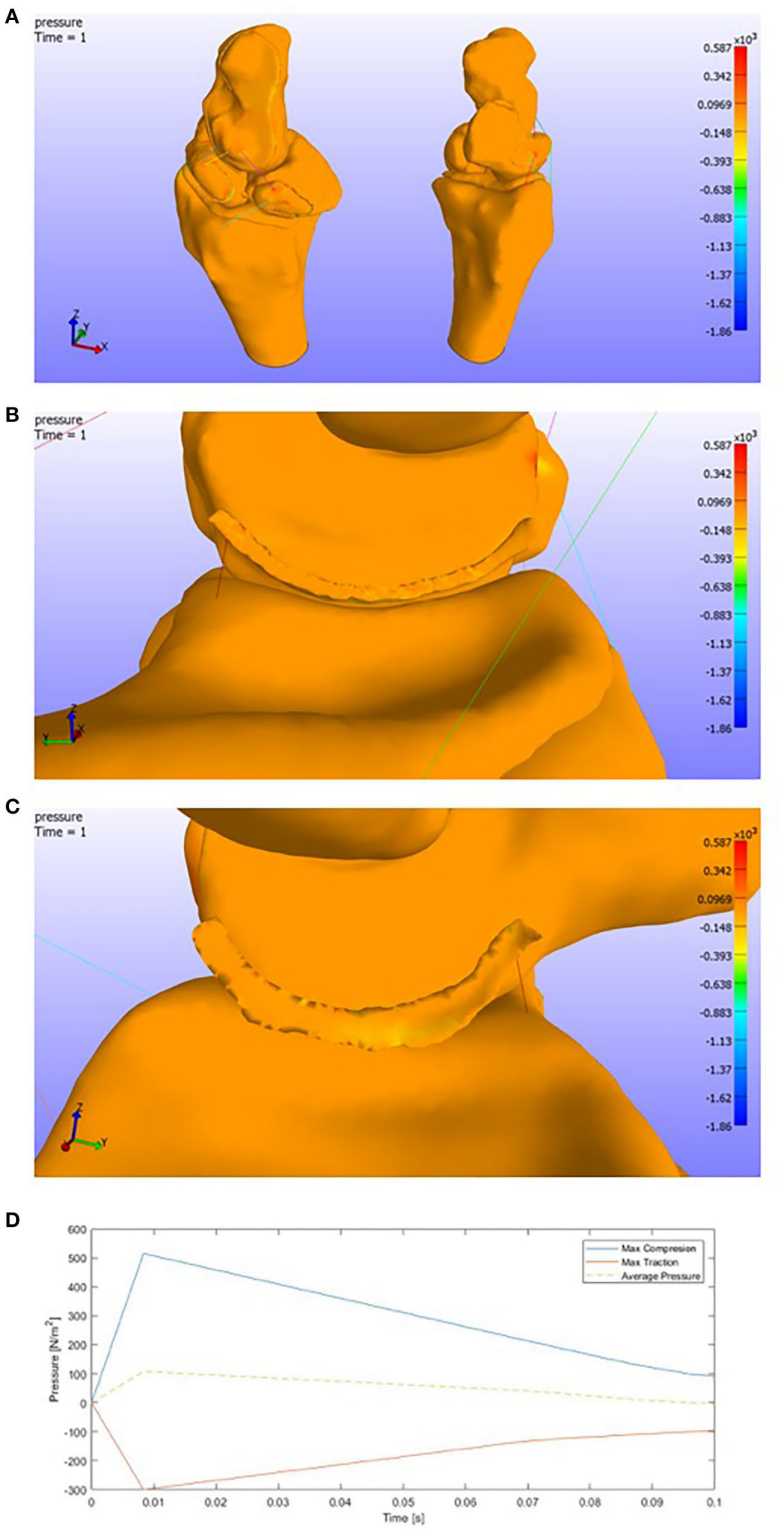
**(A)** Pressure representation on the whole structure for last time step. Extension. **(B)** Detail of the pressures on the SLIL in the scaphoid attachment. **(C)** Detail of the pressures on the SLIL in the lunate attachment. **(D)** Pressures envelope and average curve of the SLIL affected by an **Extension motion**.

As we see in Figure 9A, when we apply an extension motion to the complex, ligaments in the dorsal facet tend to make compressions in the attachment areas. The maximum pressure values (compression/traction) are:

- Max Compression = 0.58e+03 Pa
- Max Traction = 1.86e+03 Pa.

We must consider that the ligaments have been modeled by 1D spring elements, focusing all the forces in small areas.

Attending to the Scapholunate Interosseous Ligament, which is our target in this research, we can see the pressure distribution in the figures below (Figures 9B,C).

As the previous picture only shows the last time step, a graphic showing the pressures for each SLIL FE is shown below (Figure 9D). We can see that the maximum compression value is around 0.50e+03 Pa and the maximum traction value around 0.30e+03 Pa.

#### 3.6.2. Flexion

**Figure 10:**
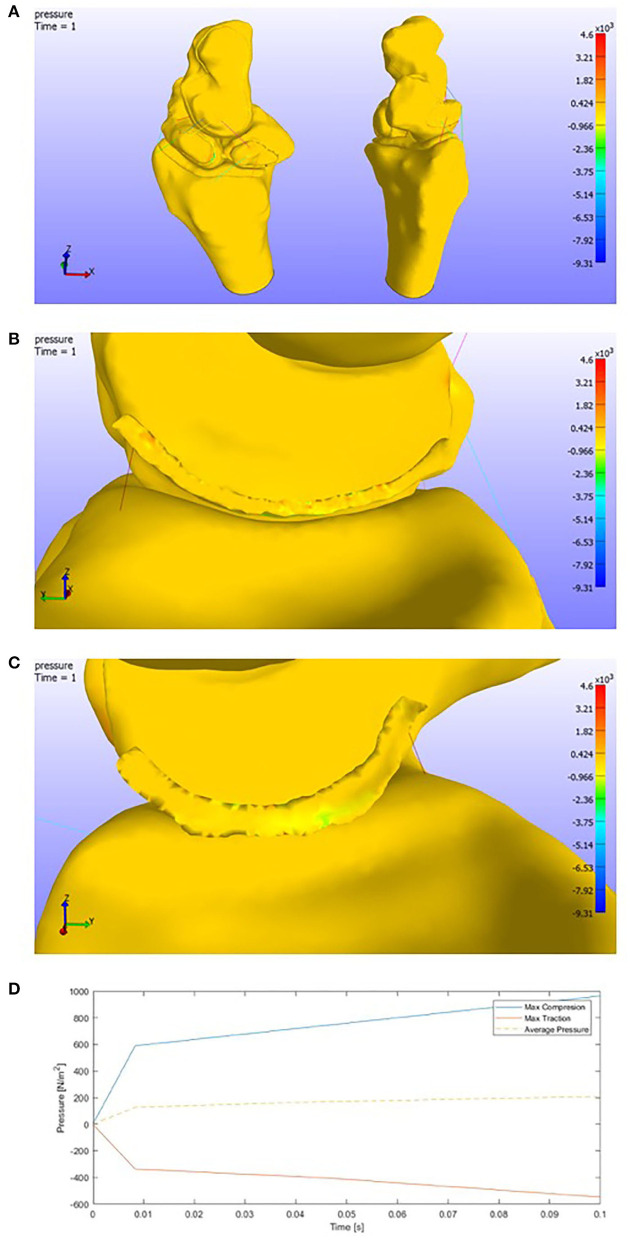
**(A)** Pressure representation on the whole structure for the last time step. Flexion. **(B)** Detail of the pressures on the SLIL in the scaphoid attachment. **(C)** Detail of the pressures on the SLIL in the lunate attachment. **(D)** Pressures envelope and average curve of the SLIL affected by a **Flexion motion**.

On the other side, in the face of a flexion motion (Figure 10A), we observe that both the compressions and tractions values increase. This increment makes sense due to the higher rotation range of motion.

The pressures in the complete model get the following maximum values:

- Max Compression = 4.61e+03 Pa
- Max Traction = 9.31e+03 Pa.

Attending to the scapholunate ligament, more tensioned areas are found than in the extension case. The maximum pressure values obtained (Figures 10B,C) are around 1.00e+03 Pa for compressions and 0.60e+03 Pa for tension.

In contrast with the extension motion, we can observe that the pressures slightly increase. We could see that the pressures tend to get lower in the final time steps in the case before (Figure 10D).

#### 3.6.3. Radial Deviation

**Figure 11:**
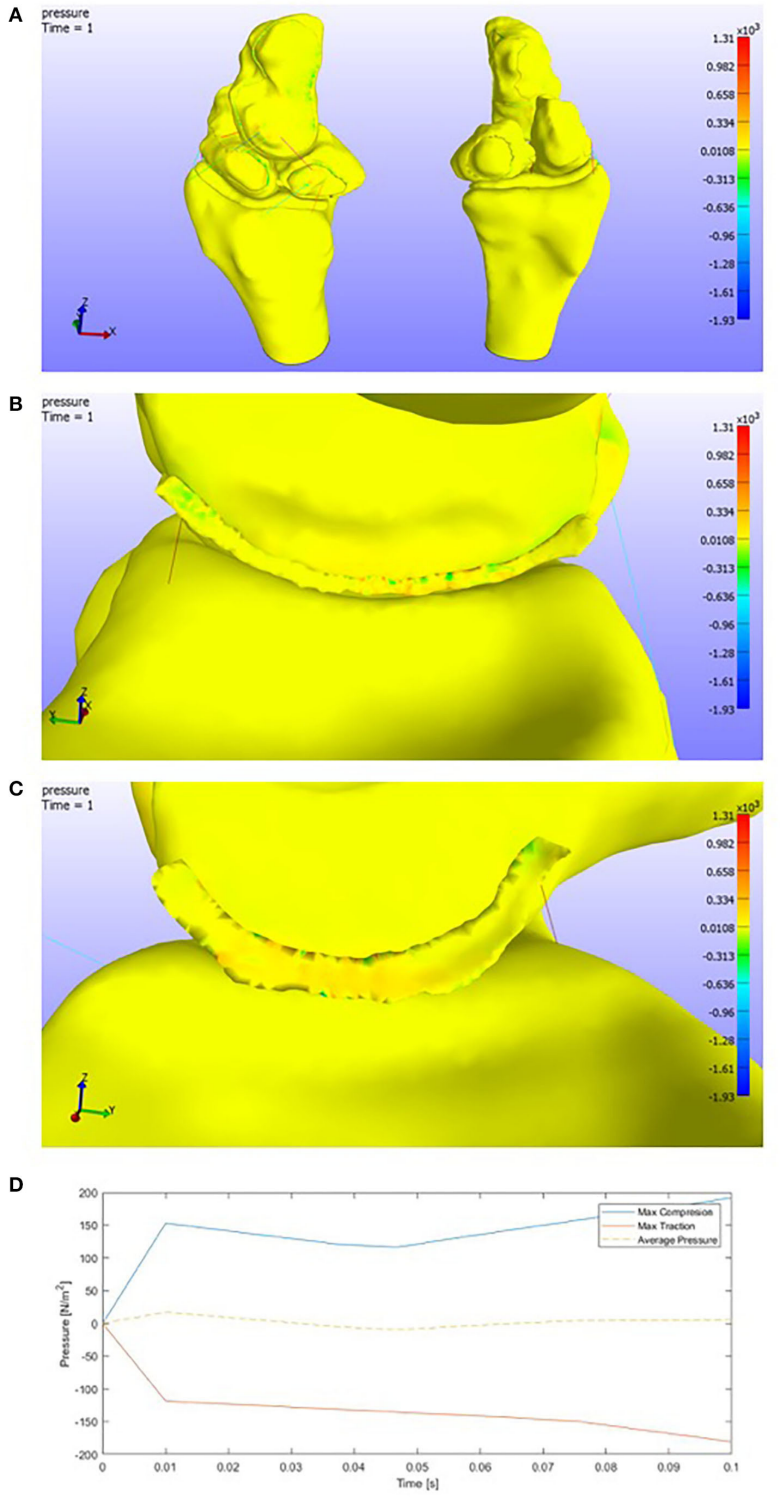
**(A)** Pressure representation on the whole structure for the last time step. **(B)** Detail of the pressures on the SLIL in the scaphoid attachment. Radial deviation. **(C)** Detail of the pressures on the SLIL in the lunate attachment. **(D)** Pressures envelope and average curve of the SLIL affected by a **Radial Deviation motion**.

Different behavior is observed by changing the plane of rotation. In this study, we can see how tractions appear in the opposite face of the deviation (ulnar area). Ligaments (spring elements) make the lunate displace with the capitate bone and both fall onto the top of the scaphoid (Figure 11A).

The maximum pressure values are:

- Max Compression: 1.31e+03 Pa
- Max Traction: 1.93e+03 Pa.

If we look at the detail of the SLIL, (Figures 11B,C), one might conclude that actually, the capitate-lunate complex leans on the scaphoid. Compressions are found in the lunate ligament facet, while tractions appear on the opposite one.

The max values, as is shown below in Figure 11D, in the SLIL are

- Max SLIL Compression: 0.20e+03 Pa
- Max SLIL Traction: 0.20e+03 Pa.

The average behavior is to increase the tension in every element. Some elements seem to lose compression after a while and commence to decrease until zero.

#### 3.6.4. Ulnar Deviation

**Figure 12:**
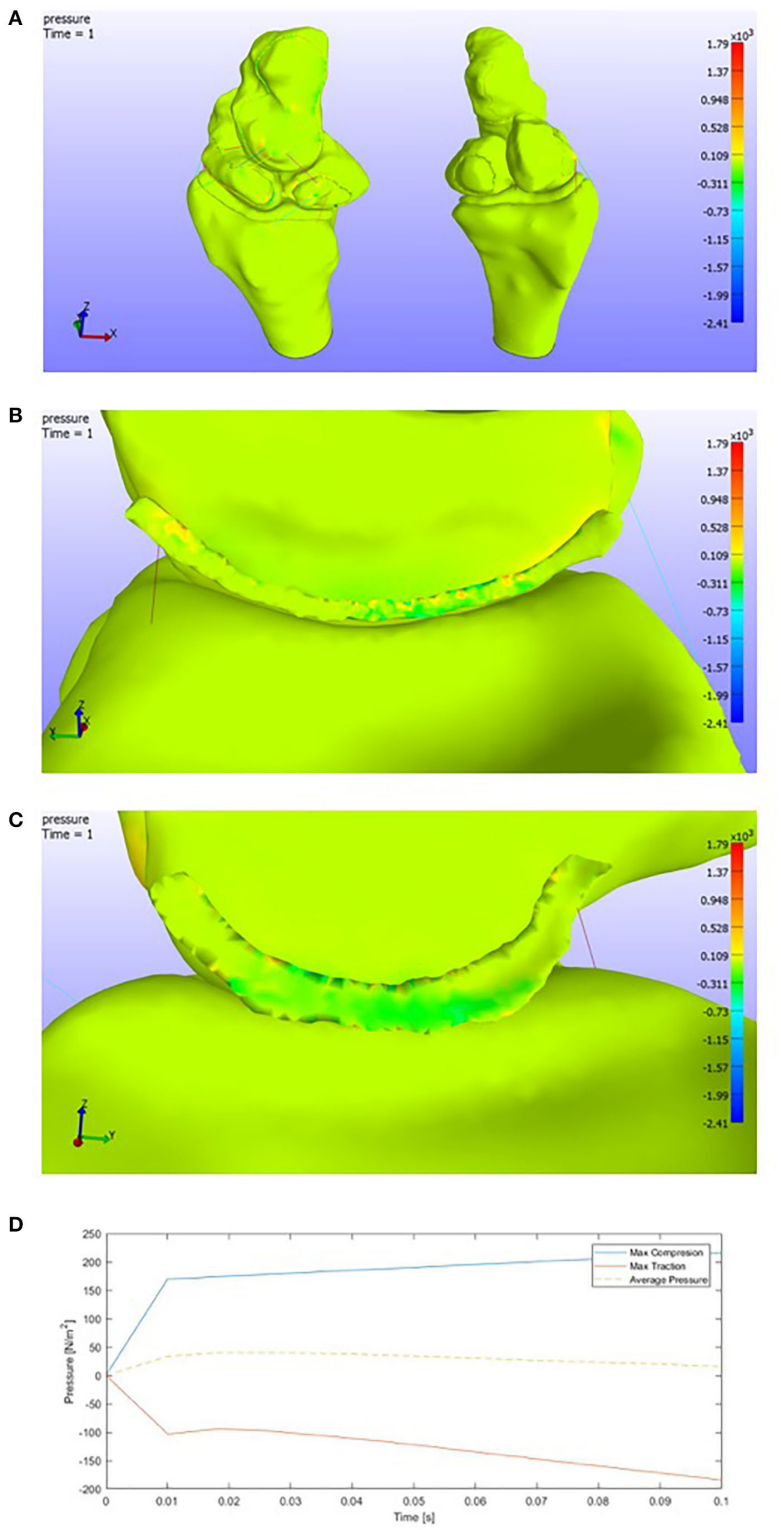
**(A)** Pressure representation on the whole structure for the last time step. Ulnar deviation. **(B)** Detail of the pressures on the SLIL in the lunate attachment. **(C)** Detail of the pressures on the SLIL in the scaphoid attachment. **(D)** Pressures envelope and average curve of the SLIL affected by an **Ulnar Deviation motion**.

Finally, in the case of ulnar deviation, we can see an opposite behavior than in the radial case, as expected. Attending to Figure 12A, we see compressions in the ulnar facet and the nearest areas to the capitate of the lunate cartilage.

If we observe Figures 12B,C, compressions are found in the scaphoid facet, tractions in the lunate one. As we have pointed previously, this is the total opposite behavior than the radial deviation. However, both positive and negative pressures are a little higher than in the previous case.

The maximum values of pressure in the scapholunate ligament (Figure 12D) are

- Max SLIL Compression: 0.22e+03 Pa
- Max SLIL Traction: 0.20e+03 Pa.

### 3.7. SLIL Break

The idea is to remove the Scapholunate ligament from the model to evaluate its stabilizing role in the bones complex.

Thus, when the software confronts this new “no-SLIL model,” it diverges. When the model diverges, it can be caused by many factors but, in this case, and, with the experience gained after all the modelization work, we can establish that this is because the carpal complex is behaving as a mechanism and it has not balanced forces.

This result leads us to the future necessity of verifying this conclusion. Hence, we plan that a “progressive SLIL break” case would be interesting to analyze. We plan future study controlling fissures in the SLIL in the face of natural wrist forces. After that, the crack would be enlarged gradually to discern when the structure starts to fail, becoming a mechanism (total structure failure).

So, although we have no quantitative results to achieve this conclusion, we can establish that the SLIL has an essential stabilizing role in the wrist motions and open the path for future studies to analyze in detail the fracture progression in the ligament and its stabilizing task in the wrist kinematics.

## 4. Discussion

As a discussion, we may comment on both the literature validations *via* results and the limitations we have found in our model. First, we can deduce, as we see in the flexion and radial deviation, the scaphoid tends to flex, leaning on the radius cartilage and forcing the lunate to move with it. This causes the ligament to withstand the loads. In the case of radial deviation, we have to add the interaction with the proper radial deviation motion, which causes a compressed area on the lunate surface. These results are near to the kinematics of the carpal row (Quigley, 2014).

Furthermore, in the face of extension and ulnar deviation motion, the lunate tends to extend in the complex due to its attachment to the triquetrum-hamate complex. Our results may validate this hypothesis. It is observed that in extension motion, the lunate facet of the scapholunate ligament gets compressed in the volar zone, which is the one compressed due to a lunate extension. Analogously, when we have an ulnar deviation motion, and in contrast with the radial deviation, compressions appear in the lunate facet (all along). The lunate extension could cause this, and it has been validated in literature (Quigley, 2014).

Otherwise, if we consider the limitations of the modelization process, they imply the exact causes of the actual wrist behavior. They consist of a complete carpal model necessary to evaluate the natural wrist behavior. In this case, although it is precise and adapts to the literature values (Crisco et al., 2011; Hammert et al., 2012), it is entirely under constrained. The behavior of the lunate is entirely contingent on the triquetrum complex, which gives it more stability. The capitate behavior is conditioned by the distal carpal row as well. This row works as a “solid structure” and joints the proximal row (our studied area) with the metacarpals. As gender and age can cause a variation in the bones properties such as Bone Mineral Density or forces between facets, and there exists a variability in bone sizes between population, a further model considering re-scalation of bones to represent different gender (de Roo et al., 2019) and ages (Kaawach et al., 2001) would be necessary.

This study comprises the first step and it needs a complete modelization of the ligaments. The ligaments and their 1D modelization tend to generate overloaded regions, particularly the ligament attachment nodes. This is not wholly accurate to the actual ligament behavior.

A future study should develop the surface of each ligament and the attachment region to each bone. Following accurate data acquisition and ranges, the main idea is to go from the 1D linear spring to describe each element to an affected area.

Therefore, a 3D modelization and hyperelastic characterization could be added. The contacts could be introduced between the complex and ligaments since some ligaments do not attach to many bones, but they have a contact relation. A dynamic study until the SLIL break can also be future study, evaluating the ultimate tensile stresses to complete the ligament's characterization.

To conclude, this wrist model has been implemented *via* hyperelasticity and biomechanical models to characterize the displacements of scapholunate. It has been developed with real human data to corroborate the values from Mohd Nazri Bajuri (2013) and calibrate our self-made axial press for future studies. The accuracy of the results gives us a quantitative limit on the kinematics of the complex, and it constitutes a keypoint at the beginning of the modelization of this ligament. The open-source coding developed is available to the research community for possible future improvements and features.

## Data Availability Statement

The link for the raw data is in the open repository zenodo (2013) under the DOI: https://doi.org/10.5281/zenodo.5607291.

## Author Contributions

The geometric characterization, CT scans, 3D reconstruction, materials modeling, and Finite Element Method (FEM) implementation were elaborated by RM, under JM and GR's supervision (Ultrasonics Lab.). The results acquisition, experimental validation, and interpretation were carried on by the Ultrasonics Lab. as well. On the other hand, the cadaveric SLIL samples extraction was made by the medical team in this manuscript (PH-C, OR, and IS-M). All authors contributed to the article and approved the submitted version.

## Funding

This research was funded by the Ministry of Education Grants DPI2017-83859-R, EQC2018-004508-P, and UNGR15-CE-3664; Ministry of Health Grants DTS15/00093 and PI16/00339; and Junta de Andalucía Grants, B-TEP-026-UGR18, IE2017-5537, P18-RT-1653, PI-0107-2017, and PIN-0030-2017.

## Conflict of Interest

The authors declare that the research was conducted in the absence of any commercial or financial relationships that could be construed as a potential conflict of interest.

## Publisher's Note

All claims expressed in this article are solely those of the authors and do not necessarily represent those of their affiliated organizations, or those of the publisher, the editors and the reviewers. Any product that may be evaluated in this article, or claim that may be made by its manufacturer, is not guaranteed or endorsed by the publisher.
